# The effect of foot massage on long-term care staff working with older people with dementia: a pilot, parallel group, randomized controlled trial

**DOI:** 10.1186/1472-6955-12-5

**Published:** 2013-02-18

**Authors:** Wendy Moyle, Marie Cooke, Siobhan T O’Dwyer, Jenny Murfield, Amy Johnston, Billy Sung

**Affiliations:** 1 Research Centre for Clinical and Community Practice Innovation, Griffith University, 170 Kessels Road, Nathan, Queensland 4111; 2 Griffith Health Institute, Griffith University, 170 Kessels Road, Nathan, Queensland, 4111; 3 NHMRC Centre of Research Excellence in Nursing Interventions for Hospital Patients, Griffith University, 170 Kessels Road, Nathan, Queensland 4111; 4 Eskitis Institute of Cell and Molecular Therapies, Griffith University, Eskitis 2 Building N75 Brisbane Innovation Park, Don Young Road, Nathan, Queensland, 4111

**Keywords:** Anxiety, Blood pressure, Care staff, Complementary and alternative medicine, Dementia, Long-term care, Massage, Mood state, Pilot, Randomized controlled trial

## Abstract

**Background:**

Caring for a person with dementia can be physically and emotionally demanding, with many long-term care facility staff experiencing increased levels of stress and burnout. Massage has been shown to be one way in which nurses’ stress can be reduced. However, no research has been conducted to explore its effectiveness for care staff working with older people with dementia in long-term care facilities.

**Methods:**

This was a pilot, parallel group, randomized controlled trial aimed at exploring feasibility for a larger randomized controlled trial. Nineteen staff, providing direct care to residents with dementia and regularly working ≥ two day-shifts a week, from one long-term care facility in Queensland (Australia), were randomized into either a foot massage intervention (*n*=9) or a silent resting control (*n*=10). Each respective session lasted for 10-min, and participants could receive up to three sessions a week, during their allocated shift, over four-weeks. At pre- and post-intervention, participants were assessed on self-report outcome measures that rated mood state and experiences of working with people with dementia. Immediately before and after each intervention/control session, participants had their blood pressure and anxiety measured. An Intention To Treat framework was applied to the analyses. Individual qualitative interviews were also undertaken to explore participants’ perceptions of the intervention.

**Results:**

The results indicate the feasibility of undertaking such a study in terms of: recruitment; the intervention; timing of intervention; and completion rates. A change in the intervention indicated the importance of a quiet, restful environment when undertaking a relaxation intervention. For the psychological measures, although there were trends indicating improvement in mood there was no significant difference between groups when comparing their pre- and post- scores. There were significant differences between groups for diastolic blood pressure (*p*= 0.04, partial η^2^=0.22) and anxiety (*p*= 0.02, partial η^2^=0.31), with the foot massage group experiencing greatest decreases immediately after the session. The qualitative interviews suggest the foot massage was well tolerated and although taking staff away from their work resulted in some participants feeling guilty about taking time out, a 10-min foot massage was feasible during a working shift.

**Conclusions:**

This pilot trial provides data to support the feasibility of the study in terms of recruitment and consent, the intervention and completion rates. Although the outcome data should be treated with caution, the pilot demonstrated the foot massage intervention showed trends in improved mood, reduced anxiety and lower blood pressure in long-term care staff working with older people with dementia. A larger study is needed to build on these promising, but preliminary, findings.

**Trial registration:**

ACTRN: ACTRN12612000659808.

## Background

There are approximately 35.6 million people with dementia worldwide and, with the aging population, this number is expected to double every 20 years [1]. Whilst the majority of older people with dementia live in their own homes, and many developed countries are now prioritizing community-provided services in national policies and initiatives, a significant number still reside in long-term care (LTC) facilities [1]. Although inherently difficult to estimate, current data suggest that approximately 53% of people residing in Australian LTC facilities have dementia [2], while about two-thirds (64%) of people with dementia live in UK care homes [3].

Care staff working in LTC facilities are often under great physical and emotional demands. Research has shown that it is time-consuming to provide care for a person with dementia [4], and that many of the behavioral and psychological symptoms of dementia, such as aggression, can lead to increased levels of stress and burnout [5], which can result in more negative attitudes and less empathy [6]. Such findings have worrying implications for the quality of care provided, particularly in terms of the increased risk of abuse and neglect [7,8]. However, there are also implications in relation to the recruitment and retention of staff, which continues to be a challenge for LTC facilities [9]. Specialized education and training of LTC staff is the likely means by which these issues will be addressed, and a recent report by the World Health Organization has advocated enhanced workforce education and training programmes on dementia and long-term care issues [1]. Effective interventions and techniques that moderate and reduce stress levels of care staff in the workplace should also play an important role.

Physiological models posit the particular effectiveness of massage therapy for inducing calming and reassuring sensations, with some evidence showing that an increased production of oxytocin positively mediates mood and social emotions [10]. Evidence from studies involving nurses, albeit limited by number and quality, suggest the potential stress alleviating effect of massage therapy when used in the clinical workplace. For example, a once-weekly 15-min back massage significantly reduced psychological symptoms of anxiety in acute care hospital nurses [11]. A combination of massage, relaxing music and aromatherapy was also shown to be effective in reducing anxiety for emergency nurses [12]. Similarly, a combination of tactile massage and hypnosis helped reduce the stress and pain levels, and increase the work ability, of short-term emergency ward nurses [13]. Finally, a pilot study, involving healthcare workers at a LTC facility for adults with severe disabilities, found that a once-weekly 20-min massage initially decreased pain severity, and showed some evidence of improving job satisfaction and morale [14]. There is an assumption that a reduction in staff stress will also influence physiological measures such as blood pressure and heart rate.

When exploring the use of massage therapy in the LTC environment specifically, research has focused solely on the effect on residents with dementia. Whilst these studies have been criticized for poor methodological quality, they provide support for the positive effects of massage as a non-pharmacological intervention on agitated behaviors in this population [15]. To our knowledge, however, comparable research has yet to be undertaken with LTC staff caring for a person with dementia. In light of this, the study described here was designed to assess the feasibility for conducting a larger, powered randomized controlled trial; and explore the potential of foot massage as a beneficial tool for the wellbeing of LTC staff providing care for people with dementia. A parallel group, randomized controlled trial was employed to ensure rigor, enable more valid and reliable conclusions to be drawn, and overcome methodological criticisms of previous research conducted in the field generally.

For the purposes of this pilot trial, it was hypothesized that when compared with the control group, participants receiving the foot massage would:

(1). demonstrate a greater reduction in negative mood as measured by the Profile of Mood States- Bipolar (POMS-Bipolar)

(2). demonstrate an increased state of relaxation, as measured by the physiologic parameters of blood pressure (BP)

(3). demonstrate a reduction in anxiety, as measured by the Faces Anxiety Scale,

(4). report an increase in positive experiences of caring for a people with dementia, as measured by the Staff Experience of Working with Demented Residents’ Questionnaire (SEWDRQ).

## Methods

### Aim

The aims of this pilot trial were to: 1. assess the feasibility of the research design including recruitment of staff, the timing of the intervention and control sessions, and completion rates; 2. compare the effect of foot massage versus a control activity of silent resting on LTC staff members’ BP (a physiological indicator of stress); anxiety; mood state; and experiences of working with people with dementia; and 3. review the trends in pre-post physiological measures to determine their use in a larger, powered randomized controlled trial.

### Design

This study was originally designed and conducted as a randomized controlled trial with cross-over, so that all participants experienced the foot massage intervention and silent resting control group. However, an unavoidable change of location in which the intervention and control activities were administered occurred just after cross-over. Participants reported to the Project Manager (PM) a strong dislike for the new room (small with no windows), including some staff reporting feeling claustrophobic, and initial inspection of results indicated a marked change in all outcome measures immediately after the room change. Because of this suspected extraneous, stressful influence on care staff, it was considered inappropriate to analyze the data after cross-over. Thus, for the purposes of analyses and reporting, the study assumes a parallel group, randomized controlled trial design.

The study was granted ethical approval by the University Human Research Ethics Committee, and was verbally endorsed by senior management at the partner aged care organization and the nominated LTC facility.

### Setting

One LTC facility, located in South-East Queensland (Australia), providing 105 beds for low (assisted), high (nursing home) and respite care to male and female residents, was nominated by the partner aged care organization as a suitable research site. This selection was based on the facility providing mainly high and dementia-specific care, and initial willingness from facility managers and care staff to participate in the research.

### Sample

This study sought a sample of 20 LTC facility care staff. In the absence of a comparable study from which to calculate a sample size, this was considered sufficient for an exploration of the feasibility of the foot massage intervention and to provide initial pilot data. For convenience five participants who were available during the one-week period following the foot massage interview were asked to participate in a short individual qualitative interview.

The two Clinical Coordinators and the Director of Nursing at the LTC facility identified potential participants and provided them with informed consent materials so they could make a decision whether to participate. Formal enrolment into the research was based on the following inclusion criteria. The member of care staff:

1. providing direct care to residents (e.g., Registered Nurses (RNs), Enrolled Nurses (ENs), Personal Care Workers (PCWs), Assistants In Nursing (AINs))

2. regularly working ≥ two day-shifts a week

3. aged ≥ 18 years

4. willing and able to complete short, self-report scales on aspects of their health, such as mood

5. willing to have their BP and anxiety measured after each foot massage and silent resting activity

6. available for work at the facility for the duration of the project, with no annual leave planned

7. providing written informed consent.

Staff were excluded from participating if they had evidence of skin infection or skin tears on one or both feet.

A member of the research team, who was blinded to the identity of eligible participants and not involved with data collection, used a computer program to undertake the permuted-block randomization process, with block sizes set at six. Participants were allocated to either the foot massage intervention or silent resting control group immediately after eligibility was determined and consent provided.

### Intervention

The foot massage intervention and silent resting control sessions were administered to participants individually, in a separate room with a closed door displaying a ‘Do not disturb’ sign. Each session lasted 10-min, and staff members could receive up to three sessions a week during their allocated shift, for four weeks. This meant that each participant could experience up to 12 sessions of either the intervention or control activity over the four-week study period (October- November 2011).

Treatment fidelity was upheld through: recruitment of two intervention Research Assistants (RA) who were practicing massage therapists and had previously been trained in the foot massage technique for another research project; comprehensive training of intervention RAs in the implementation of foot massage and silent resting, and in measuring BP and anxiety; recruitment of a Project Manager (PM) to oversee the study; a standardized procedures manual detailing the protocol for both treatment and control; and spot-checks of data collection paperwork and the massage technique at regular intervals during the study period (checked by having the intervention RA give the foot massage to the trainer).

The intervention was a foot massage delivered by one of two RAs trained by an expert certified therapist in the massage technique. In each session, participants received a standardized five-minute massage on each foot (10-min in total), involving the application of light pressure with long, gliding, rhythmical strokes of the entire foot and ankle, and toe and ankle rotation, flexion and extension [16-19]. Unscented Sorbolene (8-10mls) was applied as a lubricant for the massage.

In the silent resting control sessions participants sat silently with their eyes closed and legs slightly elevated on a beanbag for 10-min. A trained RA stayed outside the room for the 10-min period. The purpose of the silent resting condition was to help isolate whether any observed effects were because of the foot massage specifically, or because the participant received special attention and had the opportunity to be away from the work environment for a quiet time.

### Data collection

All participants were assessed on two self-report outcome measures at two main time-points during the study: pre-intervention (baseline) and post-intervention. The PM distributed the outcome measures to each participant, providing clear verbal and written instructions on how to complete each, and a contact number for any questions. Participants were asked to complete and return the measures to a locked-box located in the LTC facility within one-week:

1. *Profile of Mood States- Bipolar (POMS-Bipolar)*[20]: a 72-item measure comprising six subscales measuring both positive and negative affects (‘agreeable-hostile’; ‘composed-anxious’; ‘clearheaded-confused’; ‘confident-unsure’; ‘elated-depressed’; and ‘energetic-tired’). Participants rate each item on a four-point scale that ranges from ‘0-much unlike this’ to ‘3-much like this’. Scores greater than 50 indicate more positive mood. This version of the POMS was chosen because of its ability to assess changes in mood in non-clinical situations produced by techniques such as massage [20]. The reliability of the measure and its subscales have been reported to be good, ranging from α = 0.78 to 0.90 [21]. When used in this study, the internal consistency was excellent: α = 0.93 at both pre- and post-intervention.

2. *Staff Experience of Working with Demented Residents’ Questionnaire (SEWDRQ)*[6]: a 21-item assessment of staff experiences and satisfaction, including their relationship with other staff and relatives of residents with dementia. In the original version, statements are scored on a scale from ‘0 – not at all’ to ‘4 – extremely’, with a total score and six sub-scores summated for the member of staff’s satisfaction with: ‘feedback’; ‘care organization’; ‘one’s own expectations’; ‘patient contact’; ‘expectation of others’; and ‘the environment’. In this study, as done previously [22], the instrument was modified to include culturally appropriate wording (i.e., ‘patient’ changed to ‘resident’). Higher scores indicate greater satisfaction. This modified version of the scale has good reported internal consistency (α = 0.80) [22], and this is confirmed in the current study (pre-intervention α = 0.93 and post-intervention α = 0.87).

In addition to these outcome measures, BP (systolic and diastolic – as a physiological indicator of stress), and a brief assessment of anxiety, were measured by a trained RA immediately before and after each session. BP was measured with a Digital Wrist Blood Pressure Monitor (model #6015) from American Diagnostic Corporation, America. Anxiety was measured using the Faces Anxiety Scale [23]: a validated scale [23] that asks participants to choose from five faces showing varying levels of anxiety.

A range of demographic information was also collected about each participant pre-intervention, including: age, gender, qualifications, role, length of time working in the facility, and number of hours worked.

Qualitative interviews with a subsample of five participants were undertaken post-intervention. As a mean to assess the feasibility of conducting a larger powered trial, the interviews were designed to assess participant’s perceptions of and reactions to the intervention, and their ideas for maximizing the efficacy of the intervention. These interviews sought to explore participants’ perceptions of: the intervention; timing of the intervention; practicalities of engaging staff in such an intervention; and suggestions for improvement.

### Data analysis

All quantitative data were entered into PASW Statistics Version 20.0 (SPSS Inc., Chicago, IL, USA) for analysis. To confirm input accuracy, all entries were checked against source, and basic frequencies were run on all outcome measures and demographics to inspect the initial spread of responses, check for outliers and determine the extent of missing data. The success of the randomization process was tested on the one pre-intervention variable (age) that permitted statistical analysis (because of the small sample size), via an independent samples *t*-test.

An Intention To Treat framework was applied to the analyses, so that all randomized participants were included and the small sample size preserved. Any missing values in the outcome variables were imputed with the respective series mean produced in PASW.

Total scores were computed for the POMS-Bipolar and SEWDRQ (the two self-report measures) at pre- and post-intervention respectively. Two, repeated measure ANOVAs were then undertaken to explore whether there were any differences in the measures when comparing foot massage versus silent resting (i.e., group differences). It was considered inappropriate to analyze the subscales of the POMS-Bipolar and SEWDRQ because excessive statistical analyses of the small sample size would likely increase the risk of a Type I error.

Change scores were calculated for BP (systolic and diastolic) and anxiety for each of the 12 foot massage or silent resting sessions (i.e., post- minus pre-intervention). An overall mean change score was then calculated for the foot massage and silent resting groups (i.e., sum of change scores divided by 12). Three, one-way ANOVAs were undertaken to explore differences in the physiological effect of foot massage versus silent resting (i.e., group differences) on participants’ BP and anxiety.

All statistical tests were considered significant at the level *p* < 0.05. All statistical tests were undertaken as a means of showing trends in the pilot data and feasibility of the pre-post physiological measures.

The qualitative interviews were transcribed verbatim and then analysed using a thematic approach. The transcripts were read several times by members of the team, and emerging issues were discussed and classified into themes.

## Results

**Figure 1:**
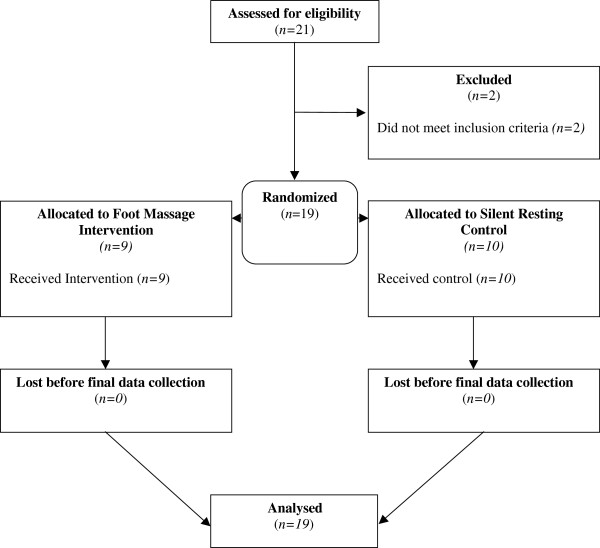
Flow of participants through study.

The recruitment of staff was successful, in that 19 members of LTC facility staff were formally enrolled into the research, randomized to intervention or control group, and analysed (see Figure 1). Random allocation to treatment group was considered successful, as there were no significant differences between groups in terms of age (*p*=0.50). There was 5.6% missing data in the study overall.

### Sample characteristics

A profile of participant characteristics is displayed in Table 1. To summarise, all participants were female, ranging in age from 23–63 years, with a mean age of 49 (σ=11.437). The majority were AINs/PCWs (*n*=16), with a Certificate in Aged Care/Nursing (*n*=14). The length of time participants’ had been employed at the LTC facility ranged from less than 12 months to more than 10 years, with one to three years being the most common (*n*=7). Participants typically worked four, seven-hour shifts per week. For those allocated to foot massage, the number of sessions they received ranged from 8 to 12, with the average number received being 11. For those allocated to quiet presence, the number of sessions ranged from 7 to 12, and the average number of sessions received was 10. This confirms the feasibility of delivering 12 sessions in the time allocated.

**Table 1:** Sample characteristics

**Characteristic**	**Sub-category**	***n***
Qualifications (*n*=17)	Certificate in Aged Care/Nursing	14
	Diploma of Nursing	2
	Bachelor of Nursing	2
	Women’s Health Education	1
Role (*n*=19)	Registered Nurse	2
	Enrolled Nurse	1
	Assistant In Nursing/Personal Care Worker	16
Time employed at the facility (*n*=19)	<1 year	3
	1-3 years	7
	4-6 years	3
	7-9 years	4
	≥10 years	2
Existing health issues (*n*=19)	Hypertension	4
	Arthritis	5
	Diabetes	1
	Skin disorder	1
	Depression	4
	Anxiety	3
Current medications (*n*=19)	Blood pressure medication	4
	Antidepressants	3
	Pain relief medication	5
Smoking status (*n*=18)	Non-smoker	12
	Current smoker	5
	Previous smoker: quit in the last five years	1

*Notes. *Total *n*=19; Participants could tick all that applied for: Qualifications; Existing Health Issues.

In terms of physical health: four participants had hypertension, for which they were taking medication; five had arthritis; four had depression (of which three were taking an antidepressant); three had anxiety; one had diabetes; and one a skin disorder. Five participants regularly took pain relief medication, and five were current smokers.

### Comparison of POMS-Bipolar and SEWDRQ, pre- and post- intervention

The reduction in negative mood hypothesis was supported in this pilot study. A repeated measures ANOVA for POMS indicated there were no significant differences in the interaction between Time (pre/post) and Group (control/intervention; p=0.666) or between the main effect of Time (p=0.875); there was however, a significant difference between the main effect of Group (p=0.03). Given that the POM means were not significantly different between the groups at baseline (independent *t*-test, p=0.061), and there is a non significant Time effect, the significant group effect appears to be spurious either due to the small sample size and/or the appropriateness of using mood as an outcome measure (See Table 2).

**Table 2:** Repeated measure ANOVAs exploring group differences in mood state and experiences of working with people with dementia

**Dependent variable**	**Total scores (σ)**						**Effect of group**		
	**Pre-intervention**			**Post-intervention**					
	Foot massage	Silent resting	Overall	Foot massage	Silent resting	Overall	F-Value	Significance (*p*=)	Partial eta squared (η^2^)
POMS-Bipolar	53.63	47.11	50.20	53.91	46.52	50.02	5.59	0.03*	0.25
	(7.49)	(6.62)	(7.61)	(7.76)	(5.02)	(7.33)			
SEWDRQ	58.56	49.48	53.78	56.11	48.75	52.24	4.24	0.06	0.20
	(10.75)	(10.14)	(11.15)	(8.22)	(6.89)	(8.25)			

*Notes. *Total *n*=19; Foot massage *n*=9; Silent resting *n*=10; *p<0.05; *σ *= Standard Deviation; *POMS-Bipolar*=Profile of Mood States- Bipolar; *SEWDRQ*=Staff Experience of Working with Demented Residents’ Questionnaire; Increases in POMS-Bipolar total scores indicate increased positive mood state; Increases in SEWDRQ total scores indicate greater satisfaction with working with people with dementia.

The hypothesis that LTC staff would be more satisfied in terms of their experiences of working with people with dementia, was not supported (*p* =0.06). A repeated measures ANOVA for SEWDRQ indicated there were no significant differences in the interaction between Time (pre/post) and Group (control/intervention; p=0.502), between the main effect of Time (p=0.220), or between the main effect of Group (p=0.055). Given that the baseline SEWDRQ means were not significantly different between the groups at baseline (independent *t*-test, p=0.075) these results indicate that the intervention had no significant effect on the mean SEWDRQ (See Table 2).

### Comparison of BP and anxiety immediately before and after intervention/control session

The hypotheses of a reduction in stress, as measured by BP, and anxiety, as measured by the Faces Anxiety Scale, were supported in this pilot study. There were statistically significant differences in diastolic BP (*F*(1,17) = 4.79, *p*= 0.04, partial η^2^=0.22) and anxiety (*F*(1,17) = 7.31, *p*= 0.02, partial η^2^=0.31) according to group, with those receiving foot massage experiencing greater decreases than their silent resting counterparts (see Table 3). This suggests that foot massage was more effective than silent resting in immediately reducing anxiety and lowering diastolic BP.

**Table 3:** One-way ANOVAs exploring group differences in blood pressure and anxiety

**Dependent variable**	**Mean change scores (σ)**			**Effect of group**		
	**Foot massage**	**Silent resting**	**Overall**	**F-Value**	**Significance (*p*=)**	**Partial eta squared (η**^**2**^**)**
Systolic Blood Pressure	−5.10	−5.88	−5.51	0.29	0.60	0.02
	(3.34)	(2.97)	(3.09)			
Diastolic Blood Pressure	−5.06	−2.25	−3.58	4.79	0.04*	0.22
	(2.46)	(3.05)	(3.07)			
Faces Anxiety Scale	−0.72	−0.30	−0.50	7.31	0.02*	0.30
	(0.42)	(0.24)	(0.40)			

*Notes. *Total *n*=19; Foot massage *n*=9; Silent resting *n*=10; *p<0.05; *σ *= Standard Deviation; Change scores were calculated for blood pressure (systolic and diastolic) and anxiety for each of the 12 ft massage or silent resting sessions (i.e., post- minus pre-intervention). An overall mean change score was then calculated for the foot massage and silent resting groups for each measure (i.e., sum of change scores divided by 12).

In terms of systolic BP, although both groups saw a non-significant reduction in mean change scores (*p* =0.60), this reduction was greatest for those in the silent resting control group (See Table 3).

The qualitative interviews found that all participants enjoyed engaging in the foot massage intervention; they described positive experiences of the massage, the break from work, and interactions with the massage staff. Participants reported feeling relaxed after the foot massage and reported that the foot massage had some impact on their work. The impact included: less pain in their feet, feelings of relaxation, increased energy, and increased ability to manage work demands. Although the foot massage was reported as a positive experience taking staff away from their work to participate in the foot massage created feelings of guilt when leaving colleagues short staffed while they were away relaxing. Participants reported potential changes to the intervention and these included changes to the location of the massage, potentially the shoulders, hands or neck at alternate sessions to help with body movement; more pressure during the massage; or more time for the massage.

## Discussion

This pilot trial demonstrated the feasibility of conducting a foot-massage intervention for staff working in LTC facilities. In terms of recruitment, although the facility was considered to be of an average size (105 beds), and the recruitment focused only on day shift staff, an adequate number of staff were willing to be involved and, thus, our estimation of the number we could recruit from one facility was accurate. Recruitment was hampered only by the trial being undertaken in the pre-holiday period when there was a large number of staff on leave. Challenges to the research design included the environmental limitations, and this demonstrates the importance of undertaking a relaxation intervention in a room where participants felt comfortable. Outcome measures were not problematic for participants to complete, however, the project manager had to remind participants to complete and return to the locked box.

Whilst the generalizability of the study’s findings are hindered by the sample size and the characteristics of the sample, which included 19 women from one LTC facility, this pilot trial provides important initial data on the feasibility and potential efficacy of foot massage for LTC facility staff working with older people with dementia. Although there were trends indicating improvement in mood the appropriateness of using POMS-Bipolar or even measuring mood in general is questionable as mood may be too malleable to measure across time. In this research mood appears to have been heavily influenced by the physical demands of the LTC workplace, whereby participant’s reported feelings of guilt at experiencing a pleasurable experience, such as that experienced from the foot massage while others were busy in the workplace. Given the effects of the workplace, and poor physical health on mood, in future research the measurement of general wellbeing may be a more appropriate measure.

The study found that members of LTC staff who received the foot massage intervention had significant decreases in diastolic BP and anxiety levels immediately after experiencing a session. These findings support those from previous massage studies involving hospital nurses and healthcare workers [11-14]. Building on these initial findings, a larger study is now needed.

The non-significant trend for all participants to be less satisfied with working with residents with dementia after the study period, particularly after experiencing the foot massage intervention, was unexpected. It is possible that the opportunity for 10-min quiet, sitting-down time and, in the case of foot massage, soothing physical touch, gave care staff an opportunity to reflect on the physical and emotional demands of the job of caring for a person with dementia, and the high-stress nature and busyness of their work environment. The time away from their work may have also raised concerns of how they were going to ‘catch-up’ on the tasks they had been unable to complete whilst participating in the study. Such reflections may have then translated into reduced satisfaction scores on the SEWDRQ. Another explanation may center on the use of the instrument chosen to assess staff work experiences, the SEWDRQ. To date, there has been only limited investigation of the scale’s psychometric properties and performance within an Australian context. However, the reliability coefficients produced in this study, and in previous work undertaken by members of the research team [22], have been good to excellent, thus offering some reassurance for suitability of the instrument. More work may be required, however, to validate this scale in an Australian setting.

The general trend for systolic and diastolic BP to immediately decrease after an intervention or control session suggests that foot massage produced a physiological relaxation response. However, the fact that decreases in diastolic BP were most marked (and statistically significant), for the foot massage group, but that the decreases in systolic BP were most marked (but non-significant) for the silent resting group, makes interpretation difficult. The research evidence on the effect of massage therapy on BP is somewhat conflicting [11,24], and the results from this pilot study reinforce the need for further exploration of whether BP is a useful and clinically meaningful indicator of the effectiveness of foot massage. At this stage, the implications of effects identified in this study are unclear and raise doubts about the feasibility of using physiological measures, such as BP, as a pre-post measure in a short intervention such as a 10-min foot massage.

Finally the qualitative interviews suggest the foot massage was well tolerated and although taking staff away from their work resulted in some participants feeling guilty about taking time out, a 10-min foot massage was feasible during a working shift.

## Conclusions

This pilot trial provides feasibility data for undertaking a larger, powered randomized controlled trial, and provides justification for the importance of environment in relaxation interventions. A larger study is now needed to determine, and isolate, the efficacy of foot massage for LTC staff.

## Abbreviations

AIN: Assistant in nursing; BP: Blood pressure; CAM: Complementary and alternative medicine; EN: Enrolled nurse; LTC: Long-term care; PCW: Personal care worker; PM: Project manager; POMS-Bipolar: Profile of mood states- bipolar; RA: Research assistant; RN: Registered nurse; SEWDRQ: Staff experience of working with demented residents’ questionnaire.

## Competing interests

The authors declare that they have no competing interests.

## Authors’ contributions

WM conceived of the study and its design, oversaw and coordinated the research, trained RAs, and assisted in drafting the manuscript. AJ and MC equally contributed by providing methodological and statistical advice, and in drafting the manuscript. SOD assisted in overseeing the study at the research site, training RAs, providing statistical advice and in drafting the manuscript. JM provided methodological and statistical advice, and took the lead in drafting the manuscript. BS undertook the statistical analysis and assisted in drafting the analysis section of the manuscript. All authors read and approved the final manuscript. No writing assistance was utilized.

## Pre-publication history

The pre-publication history for this paper can be accessed here:

http://www.biomedcentral.com/1472-6955/12/5/prepub
